# Evaluating the PRASE patient safety intervention - a multi-centre, cluster trial with a qualitative process evaluation: study protocol for a randomised controlled trial

**DOI:** 10.1186/1745-6215-15-420

**Published:** 2014-10-29

**Authors:** Laura Sheard, Jane O’Hara, Gerry Armitage, John Wright, Kim Cocks, Rosemary McEachan, Ian Watt, Rebecca Lawton, On behalf of the Yorkshire Quality & Safety Research Group

**Affiliations:** Yorkshire Quality & Safety Research Group, Bradford Institute for Health Research, Bradford Royal Infirmary, Bradford, BD9 6RJ England; Institute of Psychological Sciences, Faculty of Medicine & Health, University of Leeds, Leeds, LS2 9JT England; School of Health Studies, University of Bradford, Bradford, BD7 1DP England; York Trials Unit, Department of Health Sciences, University of York, York, YO10 5DD England; Bradford Institute for Health Research, Bradford Royal Infirmary, Bradford, BD9 6RJ England

**Keywords:** Patient safety, Safety management, Medical error, Patient participation

## Abstract

**Background:**

Estimates show that as many as one in 10 patients are harmed while receiving hospital care. Previous strategies to improve safety have focused on developing incident reporting systems and changing systems of care and professional behaviour, with little involvement of patients. The need to engage with patients about the quality and safety of their care has never been more evident with recent high profile reviews of poor hospital care all emphasising the need to develop and support better systems for capturing and responding to the patient perspective on their care. Over the past 3 years, our research team have developed, tested and refined the PRASE (Patient Reporting and Action for a Safe Environment) intervention, which gains patient feedback about quality and safety on hospital wards.

**Methods/design:**

A multi-centre, cluster, wait list design, randomised controlled trial with an embedded qualitative process evaluation. The aim is to assess the efficacy of the PRASE intervention, in achieving patient safety improvements over a 12-month period.

The trial will take place across 32 hospital wards in three NHS Hospital Trusts in the North of England. The PRASE intervention comprises two tools: (1) a 44-item questionnaire which asks patients about safety concerns and issues; and (2) a proforma for patients to report (a) any specific patient safety incidents they have been involved in or witnessed and (b) any positive experiences. These two tools then provide data which are fed back to wards in a structured feedback report. Using this report, ward staff are asked to hold action planning meetings (APMs) in order to action plan, then implement their plans in line with the issues raised by patients in order to improve patient safety and the patient experience.

The trial will be subjected to a rigorous qualitative process evaluation which will enable interpretation of the trial results. Methods: fieldworker diaries, ethnographic observation of APMs, structured interviews with APM lead and collection of key data about intervention wards. Intervention fidelity will be assessed primarily by adherence to the intervention via scoring based on an adapted framework.

**Discussion:**

This study will be one of the largest patient safety trials ever conducted, involving 32 hospital wards. The results will further understanding about how patient feedback on the safety of care can be used to improve safety at a ward level. Incorporating the ‘patient voice’ is critical if patient feedback is to be situated as an integral part of patient safety improvements.

**Trial registration:**

ISRCTN07689702, 16 Aug 2013

**Electronic supplementary material:**

The online version of this article (doi:10.1186/1745-6215-15-420) contains supplementary material, which is available to authorized users.

## Background

The public expect safety to be a priority within health services. However, estimates show that as many as one in 10 patients are harmed while receiving hospital care [1–4]. Healthcare associated harm is defined as arising from or associated with plans or actions taken during the provision of healthcare, rather than an underlying disease or injury [5]. One study of admissions analysed over a 5-year period across 10 hospitals in the Unites States [6] found that 63% of all harms were deemed preventable. The recent UK NHS Mandate [7] lists one of its core themes as: ‘treating and caring for people in a safe environment and protecting them from avoidable harm’, with the objective of reducing avoidable harm and embedding a culture of patient safety in the NHS by 2015. Accordingly, patient safety has been firmly positioned as a key NHS and government concern. Patient involvement in healthcare safety is also a policy priority with, for example, the World Health Organization’s World Alliance for Patient Safety (WHO, WAPS) citing mobilisation and empowerment of patients as one of six action areas that will be taken forward in its ‘Patients for Patient Safety’ programme [8].

Strategies to improve safety have focused on developing incident reporting systems, and changing systems of care and professional behaviour. However, there has recently been a growing interest in involving patients in safety initiatives. This reflects recent UK government policy aims for people to be generally more involved in their care [9, 10]. High profile reviews of poor UK hospital care [11–13] have all emphasised the need to develop and support better systems for capturing and responding to the patient perspective on their care. However, it is imperative that such systems are robustly developed and evaluated, and moreover, integrated into existing clinical governance systems within healthcare organisations [14].

Despite international emphasis on patient involvement in safety there is a dearth of research evidence on the acceptability of this involvement to patients and equivocal evidence to date on whether such involvement leads to improvements in safety. The evidence that exists indicates that patients are willing and able to participate in error prevention strategies [15] which have the potential to improve safety [16–19]. However, many factors hinder patient participation including acceptance of the new patient role, lack of medical knowledge, lack of confidence, co-morbidity and sociodemographic factors [20]. Thus, there is clearly a need to understand further how patients can best be involved in safety initiatives and the effectiveness of such involvement in improving safety.

Reason’s model of organisational safety [21] states that organisational accidents are a result of a number of factors including active failures on the part of the individual (for example, attentional slips, or mistakes in decision making), and ‘systems failures’ encompassing latent failures (for example, budgeting or rostering decisions) and local working conditions (for example, equipment unavailable, ward or unit understaffed). Systems failures are often referred to as ‘contributory factors’. Based on these ideas, measurement tools have been developed in high-risk industries to monitor organisations’ ‘safety health’ [22, 23]. However, currently no general means of assessing organisational safety or ‘systems’ failures exists within the NHS. Furthermore, no specific measures of organisational safety exist that ask for the views of patients, despite patients being well placed to observe the organisation of their care and the practices around them. Therefore, there is a need for reliable and valid tools that allow patients the opportunity to provide feedback on the safety of their care environment to inform local and organisational changes to improve patient safety.

Learning from error is a key element of patient safety [24], and one way to learn is through the reporting and analysis of patient safety incidents. A patient safety incident (PSI) has been defined as ‘any unintended or unexpected incident which could have or did lead to harm for one or more patients receiving NHS care’ [25]. This definition usefully encompasses a variety of situations relating to patient safety, across both adverse events themselves and near misses. Historically, efforts to learn from incident reports have been focused on staff-led reporting systems [26], with little attention paid to the potential of the patient as a valuable source of information about patient safety [18, 27–30]. Indeed, it has been argued by some authors that the patient is uniquely placed to contribute to the quality and safety of their own care [31], with recent empirical work demonstrating the feasibility and value of patient reporting [32–35]. However, no study to date has attempted to systematically develop and evaluate the most effective method of patient reporting. In addition, no study has attempted to link reporting of patient safety incidents to mainstream quality improvement mechanisms.

During the last 3 years, our research team have developed and tested the Patient Measure of Safety (PMOS) [36, 37] and the Patient Safety Incident Reporting Tool (PIRT). Together, these tools form the PRASE (Patient Reporting and Action for a Safe Environment) intervention which asks patients about the safety and quality of their care. PMOS is a 44-item questionnaire and PIRT is a reporting proforma. Patient feedback is collated into a report from which ward staff formally action plan and implement changes - in line with the issues raised by patients - with a view to improving patient safety and the patient experience. More detail about the intervention is given in the Methods section.

PRASE has recently been piloted in a trial of over 300 participants in a medium-sized hospital in the North of England. This pilot has allowed the research team to test key feasibility, usability and logistical issues. Much of the development of the intervention to date and associated work has been published elsewhere [36–38]. This paper provides the protocol for the current study which will test the PRASE intervention in a multi-centre, cluster randomised controlled trial, employing a wait list design.

## Methods/design (trial)

### Primary objective

To assess the effectiveness of the PRASE patient safety intervention, in achieving patient safety improvements over a 12-month period.

### Design

A multi-centre, cluster randomised controlled trial, randomising wards to either intervention or control in a 1:1 ratio.

### The PRASE intervention process

The PRASE intervention is designed to collect feedback from patients about the safety of their care, using robust tools developed within our team over the past 3 years. This patient feedback is then collated and presented to each ward in a formal report (a ‘feedback report’). This report then allows ward staff to understand more about how patients perceive the safety of their care on their ward, and then target improvements based on problematic areas. The philosophy of this intervention is that it is an iterative process with a cycle of measurement, feedback and change lasting for a period of 6 months. The three key stages of the process are: a) measurement, b) feedback, c) action planning and change.

a) Measurement

Over a 3- to 4-week period, an average of 25 patients per ward will be recruited to participate in the measurement phase. Each patient (deemed to have capacity to consent) will be approached, the study explained, and informed consent taken. Following this, and using a computer tablet, the research fellow or research nurse will ask the patient to complete the 44-item PMOS questionnaire, and report any safety concerns (or specific positive experiences of care) using the PIRT incident reporting tool. The 44-item questionnaire asks questions based on eight different domains which are:

- communication and team working
- organisation and care planning
- access to resources
- ward type and layout
- information flow
- staff roles and responsibilities
- equipment
- delays

An additional four questions are asked, which are described under the Secondary Outcomes section. Patients will be given a choice of whether they would prefer to self-complete the questionnaire or have it facilitated by the researcher. Patient reports of safety concerns (or positive reports) can only be recorded in a facilitated conversation with a researcher, due to the need to elicit detailed information in a specific format that needs to be typed onto the computer tablet. The PMOS questionnaire and PIRT tool are detailed in Additional file 1: Appendix 1 and Additional file 1: Appendix 2.

There are three measurement periods – at baseline, at 6 months, and a final measurement at 12 months.

b) Feedback

Following the measurement period, the information for each ward will be collated and presented to the ward in the form of a ‘Feedback Report’. This report provides a variety of different qualitative and quantitative information for staff, and has been designed and piloted by a team of patients, academics and health professionals to be as user friendly as possible. Please see Additional file 1: Appendix 3 for an example of the feedback report.

The report provides an overall ward safety profile (on pages 3 and 4) which summarises scores and number of reports (concerns or positive experiences) relating to each contributory factors domain of the PMOS questionnaire, as well as providing a breakdown of how these scores and reports relate to specific questions. PMOS questionnaire scores are shown graphically using a traffic light system to allow staff to see where they are performing well, and where improvements are necessary.

No recommendations for areas of action are suggested by the research team. The feedback report is simply a reflection of the patient’s perspective of the safety of their care. It is then up to ward staff to identify areas to target for improvement, within the next phase of the intervention.

c) Action planning and change

The next phase of the intervention is action planning, followed by implementing and monitoring changes, based on the areas identified for action in the feedback report. To undertake the action planning, we will ask participating wards to identify an Action Planning Team (APT). This team will comprise a minimum of four people who work on the ward, and ideally include both senior and more junior staff, from different professional groups. An example action planning team might include any of the following representatives: matron, consultant, ward manager/ward sister, junior doctor, staff nurse, healthcare assistant, Allied Health Professional (for example, a physiotherapist, an occupational therapist), pharmacist, ward clerk or patient representative. The APT will be responsible for receiving the feedback report, considering which area(s) should be targeted, and agreeing an action plan for improvement. In addition, the team will need to monitor the implementation of the plan. A nominated person within the APT will take responsibility for delivering the action plan.

Action Planning Meetings (APMs) will be facilitated by a senior researcher from the Quality & Safety research team. The piloting of the PRASE intervention showed that facilitation of APMs was important for generation of concrete action plans as an outcome of the action planning meeting.

Control wards will receive no intervention during the study duration. At the end of the study, control wards will receive all their feedback reports for the three time points of data collection amalgamated into one report.

### Setting and sample

This study will be undertaken within 32 hospital wards, spread across three NHS Trusts, over five different hospital sites (eight wards at a small district general hospital, 10 wards at a medium sized teaching hospital and 14 wards at a very large teaching hospital). Study participants will be patients within participating wards. An average of 25 patients within each ward will be recruited at three different time points across the study period. Each time point lasts 3 to 4 weeks and data collection occurs within the same 3- to 4-week window for all participating wards in the same Trust. The eligibility criteria for the study are given below but, briefly, the selection criteria for participation is: any patient aged 16 years or over who has capacity to give informed consent to take part. All patients who satisfy the eligibility criteria are approached and then if they agree to take part in the study and give informed consent then they are recruited into the study. Therefore, selection bias is minimal as researchers approach every patient on the ward in a stepwise fashion, unless the patient does not have mental capacity to give informed consent or they are so gravely ill or distressed to the extent that it would preclude the researcher undertaking the PMOS questionnaire with them. Recruitment of patients on a ward ceases when 25 patients have been recruited. The study will involve 800 patients at each time point and equates to a total sample of 2,400 patients. While study participants are patients, the outcome measures are at ward-level meaning that the unit of analysis is the ward.

Wards will be randomly assigned to either the intervention or control groups, on a 1:1 ratio. Randomisation will be carried out by York Trials Unit Randomisation Service (based at the University of York) using a secure computer system. All wards from each Trust will be randomised in a batch. Although there is the possibility of staff moving between control and intervention wards in a hospital, the possibility of contamination between the groups is expected to be minimal. This is because the ‘Feedback Report’ is not available to control wards and the intervention is ward-specific dependent on this. Baseline data collection will be undertaken at least 1 month before randomisation to ensure completion of data collection before knowledge of randomisation. Once baseline data have been collected in a Trust, participating wards will be randomly allocated to one of the two arms: intervention or control. Minimisation will be used to balance the groups with respect to ward specialty, average age, single/mixed sex wards and ward size. After randomisation, ‘start up’ meetings will be held with intervention wards only. Intervention wards will be asked to set up action planning meetings in order to consider their first feedback report. The above process is detailed in flow chart format in Additional file 1: Appendix 4.

### Blinding

Data collectors will be blind as to which wards are in the intervention group and which are in the control group, in order to minimise bias in data collection. Blinding of the senior researchers working on this study is not possible as they are facilitating the action planning meetings which take place with intervention wards only.

### Inclusion criteria

- Male or female
- Aged 16 years or over
- Able to give informed consent
- Minimum period of 4 h on the ward before questionnaire administered

### Exclusion criteria

- Does not have capacity to consent
- Child under the age of 16 years
- Has capacity but is too ill or distressed to take part (for example, breathlessness, pain, bleeding, immediately postoperative)
- Has already taken part in the study within the previous month

### Estimate of sample size

The study will be powered to detect a small to medium difference (effect size = 0.3) between the intervention and control groups with respect to the Patient Safety Thermometer score (See Outcome Measures for explanation of this score). A small to medium effect size seems a reasonable assumption as each ward will be focussing on developing and implementing their own action plans, tailored using their initial feedback. The intervention is therefore specific to individual wards and may not impact on all areas measured by the Patient Safety Thermometer. In order to achieve 80% power (with alpha = 0.05) with an average cluster size of 25 patients and assumed ICC of 0.05, 32 wards will be required (16 per arm). This estimate of ICC seems reasonable for a trial in secondary care with a patient reported outcome [39].

### Study period

The study will run for 16 months in each ward. We will stagger the start date for each trust, and for different wards in each participating trust, to allow effective management of the patient recruitment process.

### Outcome measures

The PRASE intervention wards receive patient feedback directly relevant to their area, and then identify areas for improvement and plan targeted changes on the basis of the areas identified. The lack of a focus on a specific safety outcome means that more generic measures of safety are necessary.

#### Primary outcome measures will be

1. Patient Safety Thermometer data. These are routinely collected hospital ward level data which all wards in England are mandated to collect on a monthly basis. It collects data about:
  - pressure ulcers
  - newly acquired venous thromboembolisms
  - catheter associated Urinary Tract Infections
  - falls

An overall measure of harm-free care is then calculated per ward. The outcome can range between 0 and 100, with higher scores indicating a higher percentage of harm-free care.

2) PMOS questionnaire domain scores. The 44-item questionnaire is scored into nine domains and one single item.

#### Secondary outcome measures

1. Three Commissioning for Quality and Innovation questions (from patients, at the end of the questionnaire) which are:
  Were you involved as much as you wanted to be in decisions about your care and treatment? (Yes, definitely/Yes, to some extent/No)
  Did you find someone on the hospital staff to talk to about your worries and fears? (Yes, definitely/Yes, to some extent/No/I had no worries or fears)
  Were you given enough privacy when discussing your condition or treatment? (Yes, always/Yes, sometimes/No).

Each question will be considered separately with the proportion of patients responding in the ‘Yes’ categories combined for analysis purposes.

2) Family & Friends question, which is: ‘How likely are you to recommend this ward to friends and family if they needed similar care or treatment?’

Patients can respond on a 5-point Likert scale (extremely likely, likely, neither likely nor unlikely, unlikely, extremely unlikely). The proportion reporting ‘Extremely likely’ and ‘likely’ will be used in the analysis.

3) Staff safety culture (Additional file 1: Appendix 5), including:
  - Ten questions from NHS national annual staff survey included items pertaining to ‘working on the ward’, ‘staff contribution to patients’, and ‘improving work practices’.
  - Four questions from the Hospital Survey of Patient Safety regarding
    perceptions of patient safety (mean of 4 items, ranging from 1.0 to 5.0 with 1 representing a low score and 5 representing a high score)
    frequency of event reporting (mean of 3 items, ranging from 1.0 to 5.0 with 1 representing a low score and 5 representing a high score)
    number of events reported in the last 12 months (1 item, categorical scale)
    a ward patient safety grade from 1 to 5 (1 = excellent, 5 = failing).

We will also seek to access from the trusts some routinely collected ward-level data from participating wards:

- Patient safety incidents for the study period will be collected using standard incident reporting by staff to the regulatory authorities. Standard reporting of patient safety incidents will be categorised into harm and no harm events.
- Complaints and comments reported to NHS Patient Advice Liaison Service (as a secondary outcome)
- Ward level covariates (staff absence/sickness rates, nurse/patient ratios and number of discharges per month) will also be collected for exploration in the analysis.

These data will be collected at baseline, 6 months and 12 months apart from the Staff Safety Culture which will be collected at baseline and 12 months.

### Management of the intervention

The agreement of senior management at the three NHS Trusts (at CEO, Chief Nurse or Medical Director level) has already been gained and participating wards identified. On the basis of previous work undertaken by our team, we realise it is important to get both ‘top-down’ agreement as well as ‘bottom-up’ engagement with ward staff, in order to secure the time, resources and motivation required to participate in such an intervention. To this end, within each participating trust, we will run a series of three, 2-h group ‘Trust debrief’ sessions with the identified APTs from the trust. The first will be a ‘start-up session’, which will run after the first phase of data collection has commenced but before any action planning meetings have begun. The primary purpose of this meeting will be to provide a more detailed briefing about the PRASE intervention, its conceptual basis, how it will work, and what is required of the APTs. A secondary purpose of this meeting will be to bring together all of the intervention wards, to identify potential barriers to the action planning process and share ideas about how best to manage them. The second meeting will be at 6 months. This ‘mid-point’ meeting will be to receive updates from each of the APTs about progress, share their success as well as trouble-shoot any problems, elicit support from senior management where interventions require resources and generally try to maintain motivation for the intervention and the study. The final ‘closing’ meeting will be for the APTs to share their experience of PRASE, discuss changes they have implemented, and allow the research team to gain contextual information about the ward which may affect the outcome measures (see process evaluation section below). Where these debriefing sessions sit in the timeframe of the trial is detailed in Additional file 1: Appendix 4.

### Recruitment and informed consent

Patients will be recruited by researchers from Bradford Teaching Hospitals NHS Foundation Trust alongside research nurses employed by the Trust in which they are collecting the data. A 1-day training course will be given to research nurses external to the core research team. Researchers will liaise with nursing staff on each ward to identify patients who have capacity and are considered well enough to take part in the research. Researchers will then approach appropriate patients to give them information about the study, both written (in the form of a participant information sheet) and verbally. The sheet will explain why the research is being conducted and what is involved in taking part. If the patient agrees to take part then informed consent will be gained and the patient will then be recruited into the trial. Patients will be informed that they can withdraw at any time without giving a reason. They will be assured that the decision to withdraw or to not take part will not affect the standard of care they receive. All additional relevant study information will be made available to participants on request (for example, copies of the research protocol). The process of recruitment and gaining informed consent has already been piloted in preliminary phases of this study and the research team are well versed in engaging and recruiting patients on hospital wards in a respectful and sensitive manner.

### Approvals process

- NHS Research Ethics Committee approval was granted by South Yorkshire REC on 15^th^ March 2013 (13/YH/0077)
- NHS Research Governance approval for:
  - Harrogate & District NHS Foundation Trust on 3^rd^May 2013
  - Calderdale & Huddersfield NHS Foundation Trust on 17^th^ May 2013
  - Leeds Teaching Hospitals NHS trust on 30^th^ May 2013
- International Standardised Randomised Controlled Trials Number from controlled-trials.com on 16^th^ August 2013

### Trial analysis

A full Statistical Analysis Plan will be written prior to any analyses. Analyses of individual level outcomes will account for clustering. For the primary outcomes, the difference between intervention groups will be estimated with 95% confidence intervals. The intervention groups will be compared with respect to the proportion of harm-free care across wards at 12 months. A linear regression model accounting for the minimisation factors and the baseline level of harm-free care will also be included as a covariate. As the outcome measure is on a ward level, no adjustment for clustering is required. The difference between the intervention groups at 12 months will be calculated with a 95% confidence interval. The standardised effect size will also be reported.

Multi-item PMOS domains will be treated as continuous data in the analysis. Linear mixed models accounting for the minimisation factors and baseline PMOS scores (ward level averages) will be used to compare the intervention groups with respect to each domain score at 12 months. Random effects will be used to account for the clustering at ward level. The difference in adjusted (least square) means will be summarised with 95% confidence intervals. Standardised effect size and ICC will also be calculated. Due to the nature of the intervention it is not possible to identify which domains are of primary interest, since the intervention across different wards may focus on improving different domains. Each domain will therefore be tested without adjustment for multiplicity. Interpretation of the *P* values will be considered carefully alongside the full details of which domain(s) the interventions in each ward were aiming to improve.

Secondary outcomes will be analysed using similar models to the primary outcomes. Ward level covariates will be added to the primary model in order to explore their influence. Continuous measures will be compared between groups using multi-level regression modelling accounting for trust/ward where required and the minimisation factors. Categorical measures will be compared between groups using logistic regression (or ordinal logistic regression for ordinal measures) adjusting for the Trust/ward where required and minimisation factors. The validity and reliability of the PMOS and domain scores will be investigated in this population using Cronbach’s alpha and known-group comparisons. All primary and secondary outcome measures will be summarised at each time point and by intervention group using summary tables or graphs/bar charts depending on the type of data.

## Methods/design (process evaluation)

The trial will be subjected to a rigorous qualitative process evaluation. Process evaluations within trials explore the: (1) implementation; (2) receipt; and (3) setting of an intervention and help in the interpretation of the outcome of results [40]. This can help improve the validity of the intervention findings alongside helping to explain specific reasons why an intervention succeeded or failed [41]. A process evaluation answers the question ‘where does the intervention work, how and why?’. A key component of a process evaluation is that of implementation ‘fidelity’ which measures the degree to which an intervention was implemented as intended [42].

The methods used to conduct a process evaluation of the trial will be:

- detailed fieldworker diaries
- in-depth ethnographic observation of the APMs
- telephone interviews with APM lead
- brief questionnaire to all staff
- collection of key information from intervention wards

The timescales for these process evaluation methods have been mapped onto the main trial timescales and detailed in Additional file 1: Appendix 6.

- Researchers will keep qualitative fieldwork diaries during the life of the trial to record their daily thoughts about what is occurring ‘on the ground’. Fieldwork diaries are informed by their regularity and personal and contemporaneous nature [43]. It is expected that researchers will write entries several times a week. The content of the diaries will focus on the culture or dynamics of a ward that may hinder or support intervention implementation alongside the fieldworker’s own personal reflection on events that have taken place [43]. Researchers will meet regularly to ensure that momentum is maintained with diary writing and to review the content to examine similarities and differences in research experiences on certain wards. Findings from these diaries will help inform how the trial has been implemented and received by the wards. In the pilot study, we are already implicitly aware of how different cultures on different wards may have influenced how the intervention was implemented and how much of this can be identified by the fieldworkers on site. Recording of this tacit knowledge will improve the validity of the intervention findings.
- One of the most important processes to occur within this trial is that of the action planning meeting (APM) where members of staff on individual wards meet to actively consider their feedback reports based on the safety data generated from patients responses. Correspondingly, APMs form the most ‘active ingredients’ of this trial as the teams digest the information from the feedback reports and consider which action plans to make. It is therefore vital that in depth ethnographic observation of these meetings forms a core part of the process evaluation if the research team are to understand how the intervention works. A research team using an intervention to improve lung cancer outcomes [42] found that observation of MDT meetings was a core element of their evaluation. The APM will be digitally recorded to allow the researchers to ‘revisit’ the meeting at a later date. Researchers will make detailed field notes straight after the APM has finished. They will start by making descriptive observations to describe ‘what, who, where and how’ [43] and then focus on processes occurring during the APM. Processes may include meanings, spaces, participation, relationships and settings [44]. A highly detailed account will be maintained. The last stage will be the researcher’s own interpretations of the observational period paying attention to the minutiae of how the ward staff have received the intervention, how they relate to each other as a team regarding the trial and how they plan action points.
- Short, structured telephone interviews will be undertaken with the nominated PRASE lead for each intervention ward involved in the APM. Participants will be asked to rate the implementation of their action plans by attributing Yes, Partial or No to each action plan. Interviewers will then probe for context as to why action plans were only partially or not implemented alongside ascertaining how successful action planning was achieved. Additional open ended questioning will primarily focus on how engaged and satisfied APM members were with the intervention. Interviews will be undertaken at 6 months and 12 months into the trial.
- At the 12-month stage, a brief questionnaire will be dovetailed into the staff safety culture survey. We will include four items to measure the ‘reach’ of the intervention, that is, how far the knowledge of the intervention has spread among staff on the ward who are not involved in the action planning group
- The collection of key information from intervention wards is an important part of this process evaluation. The research team will monitor the following:
  Did the ward staff attend an action planning meeting which was facilitated by a researcher from the PRASE team?
  How many times a ward postponed or cancelled an APM, if any?
  Were action plans made by the ward?
  Is there any documented evidence of this?
  Did the ward take part in a telephone interview about the study?
  Attendance at whole-Trust meetings (start-up, mid-point, closing)

### Process evaluation analysis

- Fieldworker diaries **-** these will be read and interrogated for common themes arising from the notes that differing researchers have made. The diaries kept during the first data collection period will be used to formulate a more prescriptive template for diary recording in the second and third data collection periods. It is likely that information will emerge about the hospital Trusts in general and then more specifically about individual wards. At this point in time, it is unknown what fieldworkers will write about and therefore diary writing during the phase one data collection period is a pilot activity in itself in order to produce a diary template for the second and third data collection periods. However, all data arising from this period will be treated as real data and included in the final analysis. We expect diary data to be subject to a thematic analysis, using a framework analysis structure [45].
- In depth ethnographic observation of APMs - An examination of the digital tape recording of the APM will focus on core themes related to action planning. These may include an examination of:
  The rationale behind the action plans which the group chose to make
  Whether ward staff chose to make systemic, upstream plans or chose ‘quick fixes’ and the context behind this
  Potential action plans which were discussed but not acted upon and the reasons why (including financial, logistic, cultural or other constraints)

These explicit data regarding what the APT discussed will be synthesised with the implicit data gathered from the facilitator’s field notes. This will give a comprehensive qualitative account of each APM. Both data sources will be synthesized and analysed, using techniques derived from adaptive theory [46]. We anticipate that this analysis may provide explanatory power for the PMOS scores alongside an understanding of wider, contextual organisational issues at play.

c) Structured interviews - An *a priori* coding framework will be devised, structured around key categorisations for why action plans were successful, partially successful or failed to be implemented. This could include concepts of autonomy, ownership or resource. However, the coding framework will also give flexibility to data that arise from the participants themselves. Similarities and differences will be interrogated between wards - particularly in the same Trust - to understand how similar action plans were implemented on some wards but not on others and the rationales behind this. Findings related to engagement and satisfaction with the intervention will point the research team towards which elements of the study ward staff may have struggled with and which elements they embraced.
d) Questionnaire to all staff - Mean scores on four Likert questionnaire items will provide an indication of the extent to which ward staff have been exposed to the different aspects of the intervention, for example, the data collection, feedback, action planning and implementation of actions. A summed score across these four items will be used as a measure of ‘reach’ in the main trial analysis.
e) Collection of key information from intervention wards - The recording of key factual information listed above will enable ‘scores’ to be derived regarding the basic fidelity of the intervention implementation for each ward (see next section for further details).

### Assessment of fidelity

An assessment of fidelity is an important component of the evaluation of complex interventions, sometimes described as the ‘black box’ of interventions [47]. Following Hasson *et al.* [41, 48] and Carroll *et al.* [49], we propose a systematic assessment of implementation fidelity of the PRASE intervention. The framework for this assessment is outlined in Additional file 1: Appendix 7, and is an adapted version of the Conceptual Framework for Implementation Fidelity [49], which has recently been used successfully in a health service intervention [48]. Three of the authors (RL, LS, JoH) agreed the key components of the intervention, and then which components were appropriate for the fidelity assessment. Some components were not deemed appropriate for a fidelity assessment as this aspect of the intervention is under the direct control of the research team (for example, ‘collection of feedback from patients about safety incidents and positive experiences of care’; see Additional file 1: Appendix 7 for other examples).

Adherence will be assessed for each intervention component, which are:

- Attendance and contribution to pan-Trust Start Up Meeting
- Holding a multi-disciplinary Action Planning Meeting to consider patient feedback about safety and quality (x 2 per intervention ward)
- Creation of action plans in response to patient feedback
- Implementation of action plans
- Attendance and contribution to pan-Trust Mid-Point Meeting
- Attendance and contribution to pan-Trust Closing Meeting

Other components of the intervention exist but these are not subject to fidelity adherence as they are researcher controlled (see Additional file 1: Appendix 7)

We will use the following criteria to assess each of the above listed intervention components:

- Content - was the intervention component implemented as planned?
- Reach - what proportion of the target group participated in this intervention component?

Following Hasson *et al.* [41, 48] and Carroll *et al.* [49], the authors also agreed which of the potential moderating factors may impact on the adherence of participating wards in each of the fidelity components. Only three potential moderating factors were selected, as the others were deemed to be under the control of the researchers within the study. The three selected were:

- Context (including management support) - what factors at political, economic, organizational and work group levels affected the implementation?
- Responsiveness - how were the participants engaged with the intervention component? How satisfied were the participants with the intervention component? How did the participants perceive the outcomes and relevance of the intervention component?
- Quality of delivery - how was the quality of delivering the intervention components?

These moderating factors will be assessed using data from the process evaluation, utilising whichever method(s) give the most accurate data to answer the relevant moderating factor question. For instance, it is anticipated that the first question under ‘responsiveness’ will be assessed via the observation of the APMs and facilitators’ notes while the second and third questions will be assessed from data gathered in the structured telephone interview stage.

Adherence to the different intervention components will be ‘scored’ on a categorical scale ranging from 0 to 3, with 0 representing ’no adherence’, 1 representing ‘some adherence’, 2 representing ‘mostly adhering’, and 3 representing ‘full adherence’. The assessment of adherence will be undertaken using the full range of information following completion of the process evaluation and largely based on satisfaction in relation to each of the moderating factors. Each intervention component will be independently scored by three members of the research team, before agreement on the final score for each ward reached through discussion and consensus. The final score will be available to be used as a covariate in the final quantitative analysis, although it is anticipated that results will be presented with and without the fidelity score.

## Discussion

This study will determine whether the PRASE patient safety intervention leads to improvements in the safety and quality of care delivered to patients in acute settings, over a 12-month period. This study will be one of the largest patient safety randomised controlled trials ever conducted, involving 32 hospital wards across three hospital Trusts in the North of England. The findings will be relevant to academics, health care professionals and policy makers alike, and add to the evidence base on the role of patients in patient safety. Specifically, it will further our understanding of how patient feedback about the safety of their care can be used to improve both cultural and objective measures of patient safety, at a ward level. This is an important step towards integrating the ‘patient voice’ into the assessment, monitoring and improvement of patient safety within hospitals. The PRASE intervention has the potential to provide hospitals with an evidence-based approach to the systematic collection of patient views about the safety of their care. Furthermore, it provides a structured approach to how staff might receive this feedback, consider it within the context of other safety intelligence, and create action plans to make targeted and patient-centred improvements on their ward.

The successful completion of this trial will present a number of challenges. First, within the current UK healthcare landscape, which is undergoing unprecedented reorganisation, and with constant pressure to ‘do more with less’, it is very possible that some staff may be reluctant to engage with the intervention. Getting healthcare professionals to see the benefits of systematically collecting patient feedback about safety will be key to ensure engagement and reduce attrition from the study. Second, a cornerstone of the intervention will be how staff receive and act on patient feedback about the safety of their care. A recent review of how staff use patient reported outcome measures (PROMs) for service improvement, suggested that one of the key issues was the extent to which healthcare professionals valued the data [50]. Specifically, across a number of studies it was suggested that a significant barrier to using PROMs was the issue of healthcare professionals not being open to receiving feedback or changing their clinical practice in light of the patient data [50]. This has been identified as a barrier for patient involvement more widely, with the ‘staff know best’ mentality sometimes inhibiting patient-centred care [51]. It is possible that the intervention components relating to advising and supporting staff about the intervention (for example, the ‘start-up’ session, facilitation of the action planning meetings), may play an important part in ameliorating these issues. The process evaluation will be able to shed light on this role after the trial is complete.

In summary, this paper has presented a protocol for a large cluster randomised controlled trial, to investigate the efficacy of using patient feedback about safety as a means of achieving patient safety improvements. It will provide some significant advancements in the emergent field of patient involvement in patient safety, as well as practical and robustly developed tools for use by health services going forward.

## Trial status

First patient recruited 7^th^ May 2013. Recruitment of patients is ongoing until September 2014.

## Electronic supplementary material

Additional file 1: **Appendix 1.** PMOS questionnaire. **Appendix 2.** PIRT tool. **Appendix 3.** Example feedback report. **Appendix 4.** Summary of trial process. **Appendix 5.** Staff safety culture questionnaire. **Appendix 6.** PRASE intervention and process evaluation: trial summary diagram. **Appendix 7.** Intervention fidelity assessment table. (DOCX 648 KB)

## Abbreviations

APM: Action Planning Meeting
APT: Action Planning Team
PIRT: Patient Incident Reporting Tool
PMOS: Patient Measure of Organisational Safety
PRASE: Patient Reporting and Action for a Safe Environment.
