# Erratum to: Evaluating the PRASE patient safety intervention - a multi-centre, cluster trial with a qualitative process evaluation: study protocol for a randomised controlled trial

**DOI:** 10.1186/s13063-016-1655-z

**Published:** 2016-12-20

**Authors:** Laura Sheard, Jane O’Hara, Gerry Armitage, John Wright, Kim Cocks, Rosemary McEachan, Ian Watt, Rebecca Lawton

**Affiliations:** 1 Yorkshire Quality & Safety Research Group, Bradford Institute for Health Research, Bradford Royal Infirmary, Bradford, England; 2 the Yorkshire Quality & Safety Research Group, Bradford, England; 3 School of Health Studies, University of Bradford, Bradford, England; 4 York Trials Unit, Department of Health Sciences, University of York, York, England; 5 Bradford Institute for Health Research, Bradford Royal Infirmary, Bradford, England; 6 Institute of Psychological Sciences, Faculty of Medicine & Health, University of Leeds, Leeds, England

## Erratum

Unfortunately, the original version of this article [1] contained an error. There was an error in the methods section. It has been corrected below:

### Published paragraph

#### Estimate of sample size

The study will be powered to detect a small to medium difference (effect size = 0.3) between the intervention and control groups with respect to the Patient Safety Thermometer score (See Outcome Measures for explanation of this score). A small to medium effect size seems a reasonable assumption as each ward will be focussing on developing and implementing their own action plans, tailored using their initial feedback. The intervention is therefore specific to individual wards and may not impact on all areas measured by the Patient Safety Thermometer. In order to achieve 80 % power (with alpha = 0.05) with an average cluster size of 25 patients and assumed ICC of 0.05, 32 wards will be required (16 per arm). This estimate of ICC seems reasonable for a trial in secondary care with a patient reported outcome [39].

### Amended paragraph

#### Estimate of sample size

The study will be powered to detect a small to medium difference (effect size = 0.3) between the intervention and control groups with respect to the PMOS score. A small to medium effect size seems a reasonable assumption as each ward will be focussing on developing and implementing their own action plans, tailored using their initial feedback. The intervention is therefore specific to individual wards and may not impact on all areas measured by the PMOS. In order to achieve 80 % power (with alpha = 0.05) with an average cluster size of 25 patients and assumed ICC of 0.05, 32 wards will be required (16 per arm). This estimate of ICC seems reasonable for a trial in secondary care with a patient reported outcome [39].
