# Supplementary material for: Evaluating the PRASE patient safety intervention - a multi-centre, cluster trial with a qualitative process evaluation: study protocol for a randomised controlled trial
Source: Trials. 2014 Oct 29;15:420. doi: 10.1186/1745-6215-15-420 (PMC4229607; doi:10.1186/1745-6215-15-420)
Supplement: Supplementary file 1 — Additional file 1: Appendix 1. PMOS questionnaire. Appendix 2. PIRT tool. Appendix 3. Example feedback report. Appendix 4. Summary of trial process. Appendix 5. Staff safety culture questionnaire. Appendix 6. PRASE intervention and process evaluation: trial summary diagram. Appendix 7. Intervention fidelity assessment table. (DOCX 648 KB) [file 13063_2014_2282_MOESM1_ESM.docx]

Appendix 4. Summary of trial process

Timeline of when research activities occur denoting months of project

Patient experience of safety measured, Phase 1

(25 patients per ward)

‘Start-up session’ with APTs (1 per Trust)

Patient experience of safety measured, Phase 2

Patient experience of safety measured, Phase 3

Final follow-up meeting held within each Trust

Wards randomised to intervention or control group

INTERVENTION GROUPS

CONTROLS

Wards identify Action Planning Teams (APTs)

Wards receive first feedback report

APTs action plan, implement and monitor changes

Wards receive second feedback report

Control wards receive feedback report with information from all three measurement periods

Wards receive final feedback report

No further action planning required

6-month ‘update’ session with APTs

Months 1 - 3

Month 3

Months 3 & 4

Months 3 & 4

Months 4 - 6

Month 5 - 8

APTs action plan, implement and monitor changes

Months 9 - 11

Months 9 -11

Months 10-12

Months 10 - 13

Months 15 - 17

Months 18 & 19

Months 18 & 19

Month 20

Appendix 1. PMOS questionnaire

**1. How likely are you to recommend this ward to your friends and family if they needed similar care or treatment?**

**Extremely Likely Neither likely Unlikely Extremely**

**likely nor unlikely unlikely**

**Please indicate your level of agreement with the following statements.**

|  | **Strongly Disagree** | **Disagree** | **Neither agree or disagree** | | **Agree** | **Strongly Agree** | **Not Applicable** | **I prefer not to answer** | **Additional Comments** |
| --- | --- | --- | --- | --- | --- | --- | --- | --- | --- |
| 1. I was always treated with dignity and respect | 1 | 2 | 3 | | 4 | 5 | N/A |  |  |
| 1. I knew who to go to if I needed to ask a question | 1 | 2 | 3 | | 4 | 5 | N/A |  |  |
| 1. The drugs I have been prescribed were always available | 1 | 2 | 3 | | 4 | 5 | N/A |  |  |
| 1. I got answers to all the questions I had about my care | 1 | 2 | 3 | | 4 | 5 | N/A |  |  |
| 1. Staff were always able to get advice from other teams about my care if needed | 1 | 2 | 3 | | 4 | 5 | N/A |  |  |
| 1. A doctor changed my plan of care and other staff didn’t know about it | 1 | 2 | 3 | | 4 | 5 | N/A |  |  |
| 1. After a shift change staff did not appear to know important information about my care | 1 | 2 | 3 | | 4 | 5 | N/A |  |  |
| 1. I knew what the different roles of the people caring for me were | 1 | 2 | 3 | | 4 | 5 | N/A |  |  |
| 1. On at least one occasion a member of staff was not able to use the necessary equipment | 1 | 2 | 3 | | 4 | 5 | N/A |  |  |
| 1. My treatment/ procedure/ operation did not always happen on time | 1 | 2 | 3 | | 4 | 5 | N/A |  |  |
| The following aspects of the ward made it difficult for **staff** to do their jobs:   1. Position of nurses’ station 2. Lighting levels 3. Clutter and untidiness 4. Lack of space | 1  1  1  1 | 2  2  2  2 | 3  3  3  3 | | 4  4  4  4 | 5  5  5  5 | N/A  N/A N/A  N/A |  |  |
| 1. I was on a ward that was not able to deal with my treatment needs | 1 | 2 | 3 | | 4 | 5 | N/A |  |  |
| 1. Staff were prompt in answering my buzzer | 1 | 2 | 3 | | 4 | 5 | N/A |  |  |
| 1. It was clear who was in charge of the staff | 1 | 2 | 3 | | 4 | 5 | N/A |  |  |
| 1. Sometimes there was no-one available to deal with aspects of my care | 1 | 2 | 3 | | 4 | 5 | N/A |  |  |
| 1. On at least one occasion a member of staff was not able to carry out a task that they should have been able to do | 1 | 2 | 3 | | 4 | 5 | N/A |  |  |
| The following aspects of the ward made it uncomfortable for me:   1. Noise levels 2. Lighting levels 3. Temperature 4. Poor cleanliness 5. Lack of space | 1  1  1  1  1 | 2  2  2  2  2 | 3  3  3  3  3 | | 4  4  4  4  4 | 5  5  5  5  5 | N/A  N/A  N/A  N/A  N/A |  |  |
| 1. Other - Please specify |  | | |  | | | | | |
| 1. I felt that the attitude of staff towards me was poor | 1 | 2 | 3 | | 4 | 5 | N/A |  |  |
| 1. I knew which consultant was in charge of my care | 1 | 2 | 3 | | 4 | 5 | N/A |  | **Please turn over.** |
| 1. Staff always seemed to know what they were meant to be doing | 1 | 2 | 3 | | 4 | 5 | N/A |  |  |
| 1. There were enough staff on the ward to get things done on time | 1 | 2 | 3 | | 4 | 5 | N/A |  |  |
| 1. Staff gave me different information about my care | 1 | 2 | 3 | | 4 | 5 | N/A |  |  |
| 1. Staff/patients waited a long time for porters to arrive | 1 | 2 | 3 | | 4 | 5 | N/A |  |  |
| 1. Staff did not work together as a team here | 1 | 2 | 3 | | 4 | 5 | N/A |  |  |
| 1. There was equipment that staff found difficult to use (e.g. monitoring equipment, beds, hoists) | 1 | 2 | 3 | | 4 | 5 | N/A |  |  |
|  |  | | |  | | | | | |
| 1. I have needed treatment and there has been no-one available who was trained to do it | 1 | 2 | 3 | | 4 | 5 | N/A |  |  |
| 1. Staff were kept waiting for my test results | 1 | 2 | 3 | | 4 | 5 | N/A |  | **Please turn over.** |
| 1. Nurses were always able to get help from other staff when they asked for it | 1 | 2 | 3 | | 4 | 5 | N/A |  |  |
| 1. Equipment needed for my care was always working properly | 1 | 2 | 3 | | 4 | 5 | N/A |  |  |
|  |  | | |  | | | | | |
| 1. I always knew which nurse was responsible for my care | 1 | 2 | 3 | | 4 | 5 | N/A |  |  |
| 1. Equipment and supplies were not always available when needed (e.g. hoists, bed pans, drugs) | 1 | 2 | 3 | | 4 | 5 | N/A |  |  |
|  |  | | |  | | | | | |
| 1. Staff always agreed about my treatment/care | 1 | 2 | 3 | | 4 | 5 | N/A |  |  |
| 1. I always felt staff listened to me about my concerns | 1 | 2 | 3 | | 4 | 5 | N/A |  |  |
| 1. Staff seemed to struggle to get help when they needed it | 1 | 2 | 3 | | 4 | 5 | N/A |  | **Please turn over.** |
| 1. When staff talked about my care with others the information they shared was correct | 1 | 2 | 3 | | 4 | 5 | N/A |  |  |
| 1. Information about me that my healthcare team needed was always available (e.g. drug charts, medical notes, test results) | 1 | 2 | 3 | | 4 | 5 | N/A |  |  |

1. **Were you involved as much as you wanted to be in decisions about your care and treatment?**

Yes, definitely Yes, to some extent No

1. **Did you find someone on the hospital staff to talk to about your worries and fears?**

Yes, definitely Yes, to some extent No I had no worries or fears

1. **Were you given enough privacy when discussing your condition or treatment?**

Yes, always Yes, sometimes No

Appendix 2. PIRT tool

1) Date of report: ___________

2) We would like to know about the things that may have concerned you about your care, or the care of others, during this hospital stay. Please tell us what happened with your concern or experience, in as much detail as you can?

3) Why do you feel this was a ‘safety concern’ for you?

4) What do you think could be done to stop this from happening again to you or other patients, in the future?

5) On a scale of 1 to 10 how serious do you think your ‘safety concern’ was?

| 1 | 2 | | 3 | 4 | 5 | 6 | 7 | 8 | 9 | | 10 |  |
| --- | --- | --- | --- | --- | --- | --- | --- | --- | --- | --- | --- | --- |
| Not serious  at all | |  |  |  |  |  |  |  |  | Extremely serious | | |

6) Do you think it would have been possible to have stopped your experience from happening?

Definitely Probably Probably Definitely Don’t know

yes yes not not

7) Which category best describes your safety concern or experience?

1. Communication and teamwork
2. Access to resources
3. Information flow

1. Medication
2. Organisation and care planning
3. Ward type and layout
4. Staff roles and responsibilities
5. Equipment
6. Other

Appendix 3. Example feedback report

**PRASE SUMMARY FEEDBACK REPORT: WARD A**

| Which ward is this feedback report about? | A |
| --- | --- |
| When did data collection start and finish? | 8^th^ to 24^th^ August 2012 |
| How many patients did our researchers speak to? | 21 (10 male, 11 female) |
| What was the total number of reported safety concerns? | 12 |
| What was the total number of patient reported positive experiences | 7 |
| Who should I contact if I have any queries about this report? | Dr Jane O’Hara (Senior Research Fellow): [Jane.O’Hara@bthft.nhs.uk](mailto:jane.ward@bthft.nhs.uk), 01274 383692  Dr Laura Sheard (Senior Research Fellow) [Laura.Sheard@bthft.nhs.uk](mailto:Laura.Sheard@bthft.nhs.uk) 01274 382828 |

**WHAT DID WE ASK PATIENTS ABOUT?**

| Communication and team working | The effective exchange and sharing of information between staff, patients and departments including written and verbal communication systems. Team working of professionals within a group. |
| --- | --- |
| Organisation and care planning | Factors related to the care plan, and the availability of resources for the care plan. |
| Access to resources | The availability of experienced staff, equipment and external resources. |
| Ward type and layout | The patients’ experience of the ward environment. |
| Information flow | The availability of information about the patient and transfer of information between staff |
| Staff roles and responsibilities | Clear supervision and lines of accountability for staff. |
| Staff training | Staff competency and ability to perform role at appropriate grade |
| Equipment | The design and functioning of equipment on the ward. |
| Delays | Delays relating to a specific procedure, or to general aspects of care. |

**HOW TO INTERPRET THE SUMMARY REPORT AND DETAILED REPORT**

**Please read this section – it outlines how to understand the data that makes up the rest of the report**

- Researchers came to your ward and asked patients about the quality and safety of their care. We facilitated the patients completing a) a 44 item questionnaire based on the factors known to contribute to patient safety incidents (PMOS - Patient Measure of Safety) and b) a tool to invite patients to report any safety concerns or experiences, or positive experiences of care (PIRT – Patient Incident Reporting Tool).
- This report is made up of a Summary Report (pages 1, 3 and 4) and a Detailed Report (page 5 to end). The Summary Report gives you information at a glance about the patient feedback regarding your ward. The Detailed Report allows you to look at individual questions to ascertain exactly where patient safety concerns or positive comments are being reported.
- All graphs are displayed using a traffic light system - dark red indicates the most negative response possible, dark green indicates the most positive response possible.
- The answers participants gave are shown in percentages of the total valid response for each of the nine areas of questioning. We have not included ‘not applicable’ options therefore the total number of responses for each question may vary.
- The free text comments you will read in the Detailed Report arise from comments that patients made about the care of themselves or others in hospital - both positive and negative. When a concern was considered to be significant to the patient or related to a specific incident, the Patient Incident Reporting Tool was used to document the report. Patient reports of safety concerns or experiences should generally be given more attention than general patient comments. The latter were experiences related to specific questions but that the patient did not believe warranted specifically reporting as an ‘incident’ to our researchers. It is clearly marked which are general comments, and which are patient reported safety concerns.
- Both patient reported safety concerns and general comments are usually recorded as a third person statement agreed between the researcher and patient.
- We ask the patient to rate the severity (from their perspective) of their safety concern on a scale of 1 to 10, where 1 is not serious at all and 10 is extremely serious.
- We also ask them to rate the preventability of the incident (from their perspective) on a 5-point scale ranging from ‘definitely preventable’ to ‘definitely not preventable ‘
- Please consider patient reported safety concerns in conjunction with the overall traffic light scores and not in isolation. It is important to look at the scores which the whole sample of patients reported as well as the verbatim safety concern reporting.
- You will see that patient reported safety concerns are mostly linked to a question in order to give contextual data for the scores that you are seeing. But when a patient reports a concern that is not linked to a specific question then this is shown at the end of the report.

**SUMMARY REPORT - Overall safety profile from questionnaire responses**


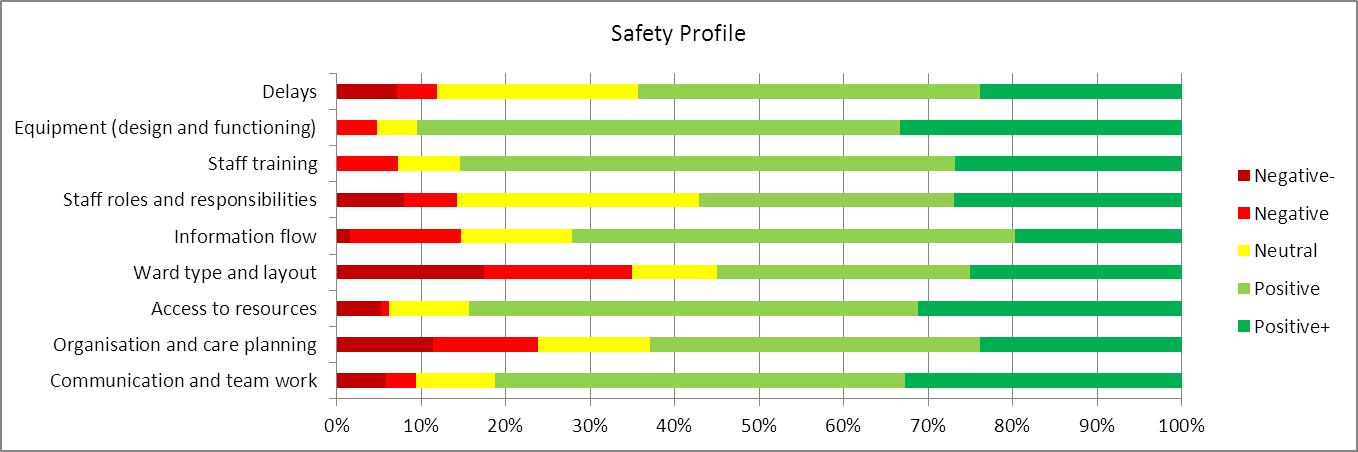


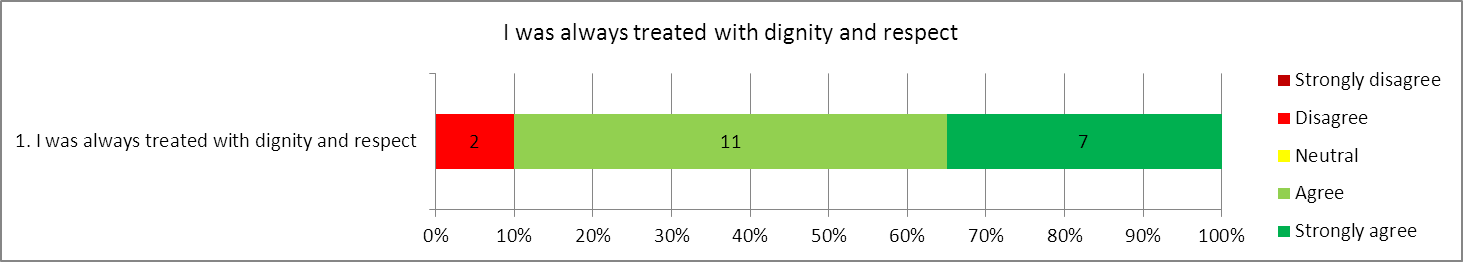


**SUMMARY REPORT – Patient reported safety concerns and positive experiences**

**NB – The numbers here represent the frequency of patient reported safety concerns and positive comments for each area of questioning (does not include responses unrelated to a specific question)**

**DETAILED REPORT**

1. **Communication and team working**


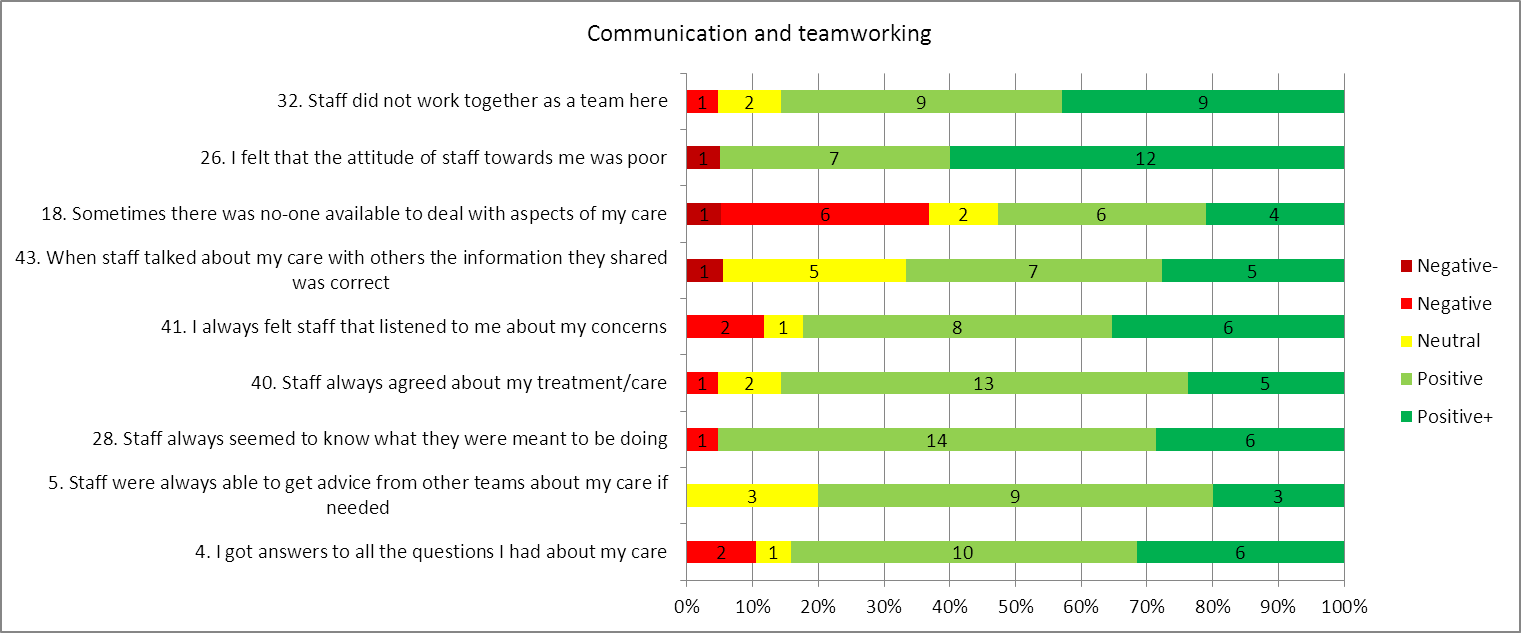


No patients reported a safety concern or experience related to any of the above questions

**What other general comments did patients make in relation to the questions about communication and team working?**

| **Question No.** | **Question Text** | **Patient Comment** |
| --- | --- | --- |
| 4 | I got answers to all the questions I had about my care | *I did not know who to ask or they were not always available. I am still not sure what they have done to me. I would have liked to see the surgeon.* |
| 5 | Staff were always able to get advice from other teams about my care if needed | *Physios particularly good* |
|  |  | *Information from GP about medication.* |
| 43 | When staff talked about my care with others the information they shared was correct | *On my sheet my birthday has been wrong for a few days. It says the 21st but It’s the 31st but they always agreed with me when I said the 31st.* |
| 18 | Sometimes there was no-one available to deal with aspects of my care | *Yes but not for long.* |
|  |  | *Sometimes, because the staff are busy.* |
|  |  | *Not me but noticed adjacent patient on the commode for a long time. When you've had an accident and feeling unwell, it is something you don't need but impossible for staff to be on the spot for everyone.* |
| 26 | I felt that the attitude of staff towards me was poor | *On a day shift their attitude is bad not just towards me but towards everyone. On the day shift I hate waking up in the morning.* |
|  |  | *Now and again just a little grumpy.* |

**What positive comments did patients make when asked about communication and team working?**

| **Question No.** | **Question Text** | **What happened / why did it happen?** |
| --- | --- | --- |
| 26 | I felt that the attitude of staff towards me was poor | *Very friendly and helpful. Cannot praise the staff enough.* |
| 32 | Staff did not work together as a team here | *They always work as a team here.* |

**2. Organisation and care planning**


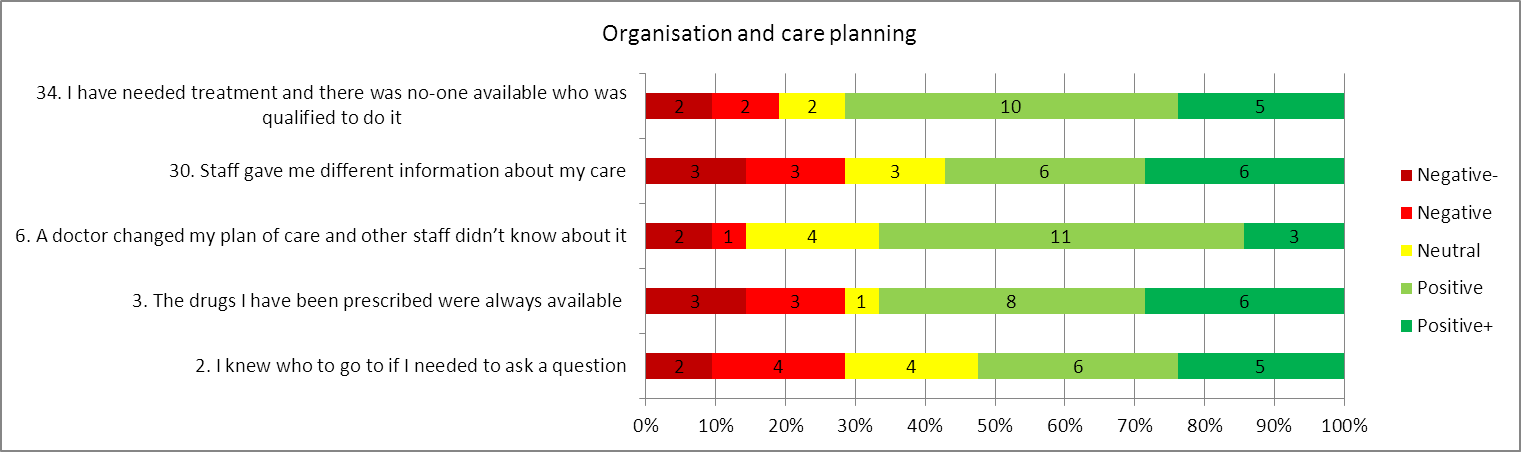


**What patient reported safety concerns were linked to questions about organisation and care planning?**

| **Question No.** | **Question Text** | **What happened / why did it happen?** | **What can be done to prevent it happening again?** | **Patient-rated preventability** | **Patient-rated severity** |
| --- | --- | --- | --- | --- | --- |
| 3 | The drugs I have been prescribed were always available | *When I first had my operation I was in quite a lot of pain, and I had to wait quite a while for painkillers. No-one's fault - the notes hadn't come up somewhere else and the nurses needed to look at these first and consult the doctor, the doctor was in A&E so had to wait 2 hours. I was in agony but it was not their fault.* | *There should be further staff on to deal with all the paperwork.* | Probably yes | 5 |
| 30 | Staff gave me different information about my care | *When I started bleeding it was a lot to begin with, I think I bled all night. One of the doctors said 'I think one of the wires might have hit an artery' and I thought 'oh great' and the other doctor said 'no, no, all that's happened is it has touched a vein and it will eventually close' which I think it has. It wasn't made clear to me what had happened to cause the bleeding. And a nurse was calling 'oh you're bleeding, you're bleeding' and that was all I needed to hear. It would have been useful to know exactly what had happened. I didn't know what had happened which was worrying.* | *If they have more time for an individual, it could be prevented, but I have no idea if that can be rectified.* | Probably yes | 7 |

**What other general comments did patients make in relation to the questions about organisation and care planning?**

| **Question No.** | **Question Text** | **Patient Comment** |
| --- | --- | --- |
| 2 | I knew who to go to if I needed to ask a question | *I would ask anyone and they would point me in the right direction.* |
| 3 | The drugs I have been prescribed were always available | *There is a drug that they have run out of, hopefully it will arrive today for me.* |
| 30 | Staff gave me different information about my care | *Staff gave me no information about my care.* |
|  |  | *I was told by the doctor that I was going to be taken down for surgery but I don't know what’s happening now. They are telling me that there is damage to my pelvis so I don't know what’s happening.* |
|  |  | *With regards to what will happen at home, it is not clear.* |
| 34 | I have needed treatment and there was no-one available who was qualified to do it | *Only because busy - just had to wait.* |

No specific positive comments were received that related to the above questions

**3. Access to resources**

**
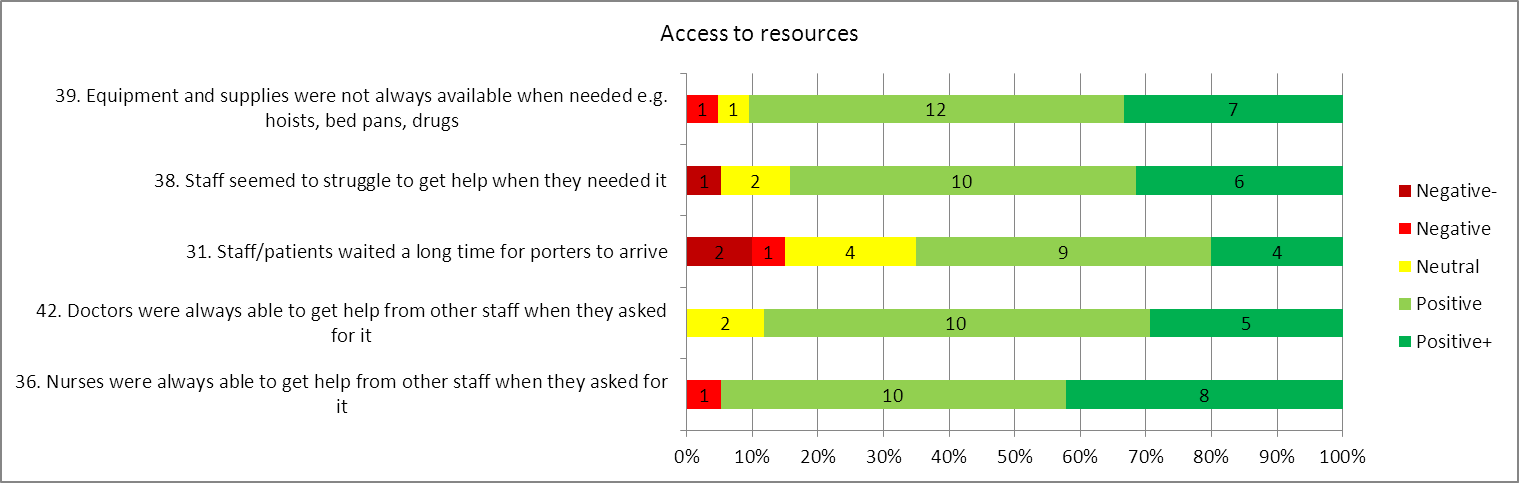
**

No patients reported a safety concern or experience related to any of the above questions

No specific general comments were received that related to the above questions

**What positive comments did patients make when asked about access to resources?**

| **Question No.** | **Question Text** | **Patient Comment** |
| --- | --- | --- |
| 31 | Staff/patients waited a long time for porters to arrive | *They're all very polite and nice and will help as much as possible.* |

**4. Ward type and layout**


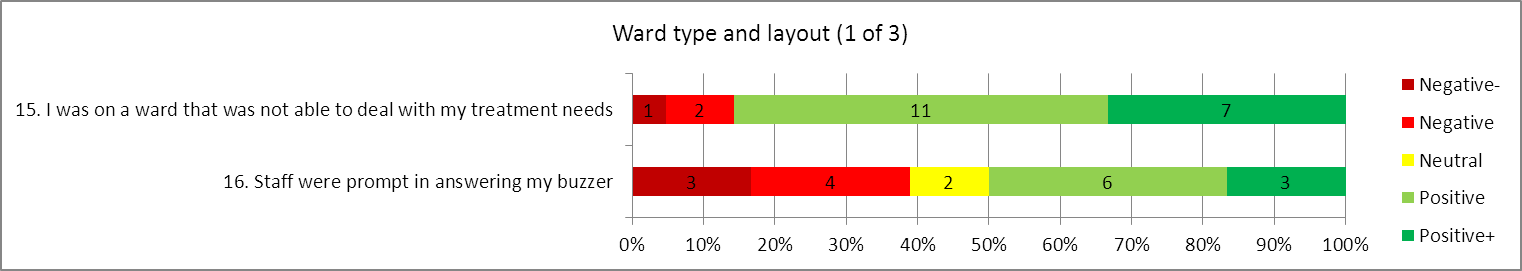


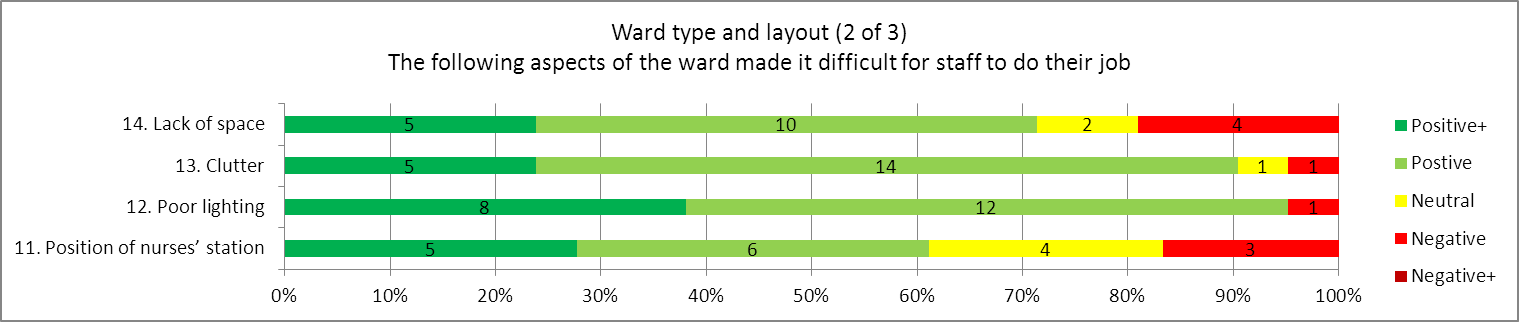


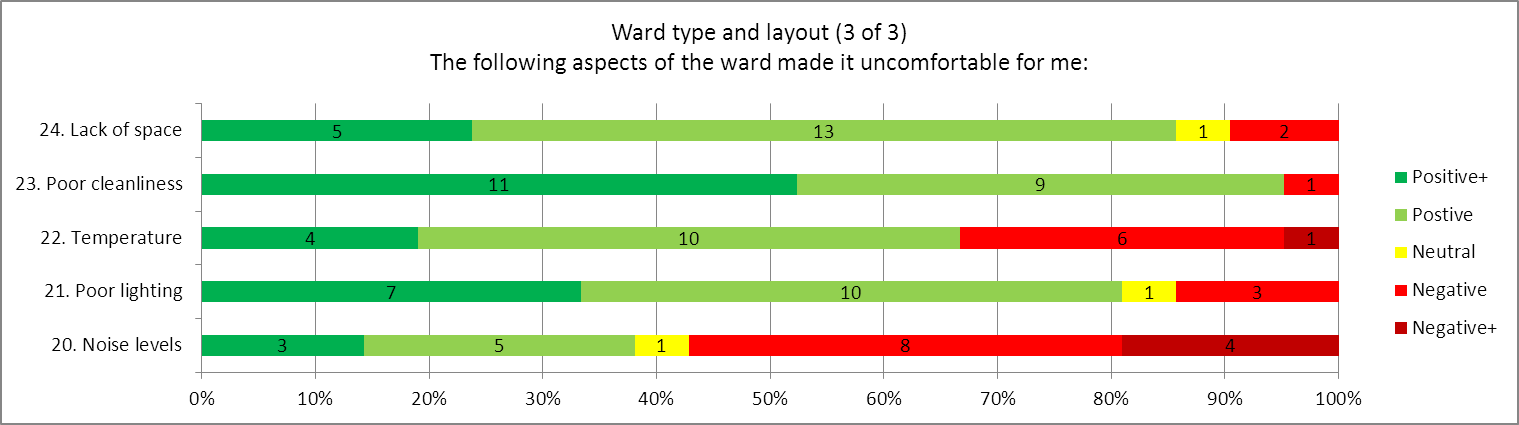


**What patient reported safety concerns were linked to questions about ward type and layout?**

| **Question No.** | **Question Text** | **What happened / why did it happen?** | **What can be done to prevent it happening again?** | **Patient-rated preventability** | **Patient-rated severity** |
| --- | --- | --- | --- | --- | --- |
| 16 | Staff were prompt in answering my buzzer | *Waiting for buzzer - needing the commode.* | *Not sure as they have so many calls from others* | Don’t know | 5 |
|  |  | *Patient pressed the buzzer and no one came for a while. Unable to get out of bed unaided. This meant patient had to lay in their own urine for quite a while until a staff member was available. It was very embarrassing for the patient. The patient spoke to a member of staff about it who was passing, and they said that it was not their job. Very embarrassing.* | *Staff should answer the buzzer quicker.* | Probably yes | 7 |
|  |  | *I pressed a buzzer but it was ages before she came. I needed to go for a pee and I couldn't get up so I needed a bottle.* | *Yes of course.* | Definitely yes | 5 |
| 20 | The following aspects of the ward made it uncomfortable for me: Noise levels | *The doors bang when they shut - like a clap of thunder, particularly at night. It has woken me up and can keep others awake too.* | *Could there be some sort of device on doors which could help them shut properly?* | Probably yes | 3 |
|  |  | *Phone ringing all the time without being answered.* | *Don't know because they can be too busy to answer it.* | Don’t know | 6 |
|  |  | *Noise levels at night - other patients can be noisy.* | *Nothing obvious.* | Probably not | 4 |

**What other general comments did patients make in relation to the questions about ward type and layout?**

| **Question No.** | **Question Text** | **Patient Comment** |
| --- | --- | --- |
| 16 | Staff were prompt in answering my buzzer | *Not always, depending on time of day - particularly evenings.* |
|  |  | *Sometimes it takes a while because they are busy.* |
|  |  | *As prompt as can be but very busy.* |
|  |  | *Prompt as they could be, as really busy ward.* |
|  |  | *Not their fault but often involved in something else they can't get away from. They come as soon as they can.* |
|  |  | *This is mainly at night that I have been kept waiting a long time for someone to come.* |
| 13 | The following aspects of the ward made it difficult for staff to do their jobs: Clutter | *Zimmer and extra items like footstools make it a little cluttered.* |
| 14 | The following aspects of the ward made it difficult for staff to do their jobs: Lack of space | *Space-wise difficult e.g. getting a patient onto a commode with a curtain round.* |
| 20 | The following aspects of the ward made it uncomfortable for me: Noise levels | *The pinging from the buzzers was irritating.* |
|  |  | *But because I cannot hear anything I don’t hear the noise. The other patients have told me it’s noisy on the night.* |
|  |  | *Noisy during the day. It is quieter during the night.* |
|  |  | *It’s very noisy on here - the main doors are always banging* |
|  |  | *Mainly at night, some of the staff talk very loudly.* |
|  |  | *One patient is particularly noisy. She screamed all night. Not sure what could be done about this.* |
| 21 | The following aspects of the ward made it uncomfortable for me: Poor lighting | *It is too bright with the lights on if the sun is shining through the window as well.* |
|  |  | *Too bright in the day.* |
| 24 | The following aspects of the ward made it uncomfortable for me: Lack of space | *It is difficult to get out of bed to my Zimmer frame because of my table.* |

No specific positive comments were received that related to the above questions

**5. Information flow**


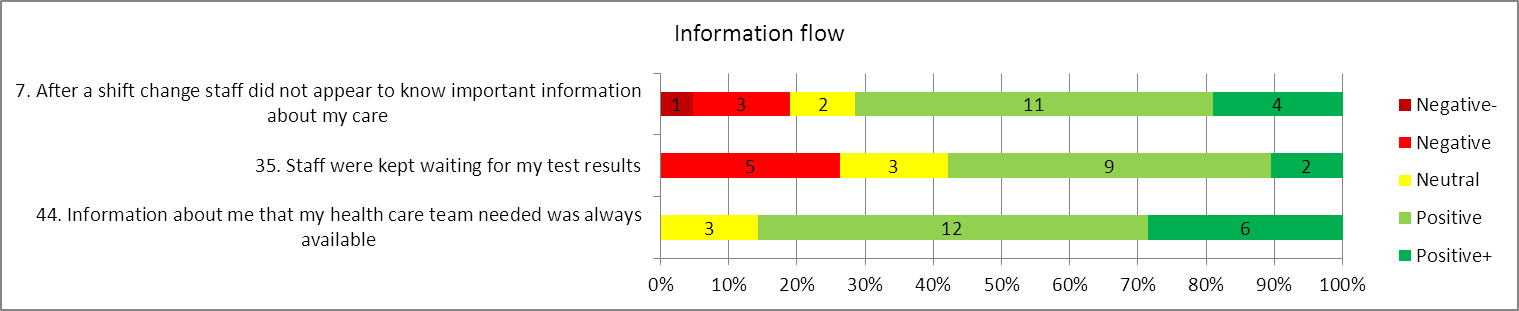


No patients reported a safety concern or experience related to any of the above questions

No specific general comments were received that related to the above questions

No specific positive comments were received that related to the above questions

**6. Staff roles and responsibilities**

**
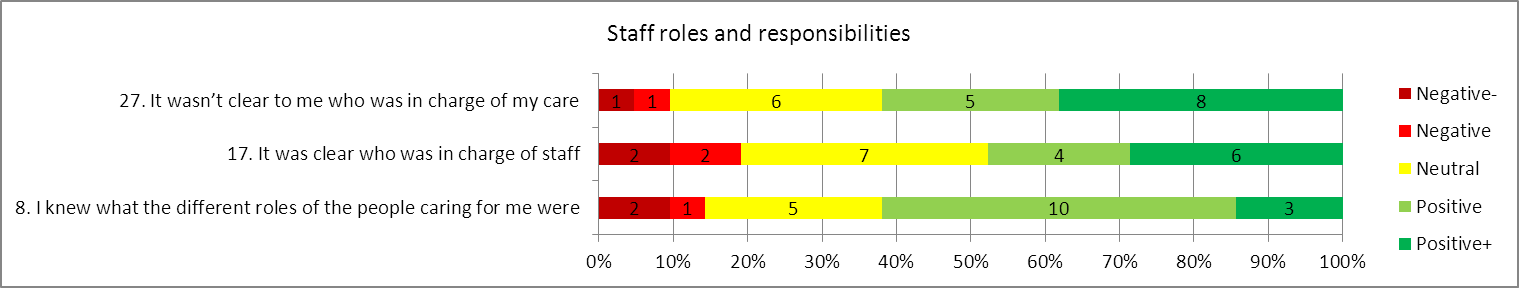
**

No patients reported a safety concern or experience related to any of the above questions

**What other general comments did patients make in relation to the questions about staff roles and responsibilities?**

| **Question No.** | **Question Text** | **Patient Comment** |
| --- | --- | --- |
| 8 | I knew what the different roles of the people caring for me were | *Sometimes - it can be difficult but they do always explain who they are. If it is a very elderly patient it must be hard for them to remember all the names and roles of staff.* |
|  |  | *I didn't know what the different uniforms were and how they relate to nursing qualifications.* |

**7. Staff training**

No specific positive comments were received that related to the above questions


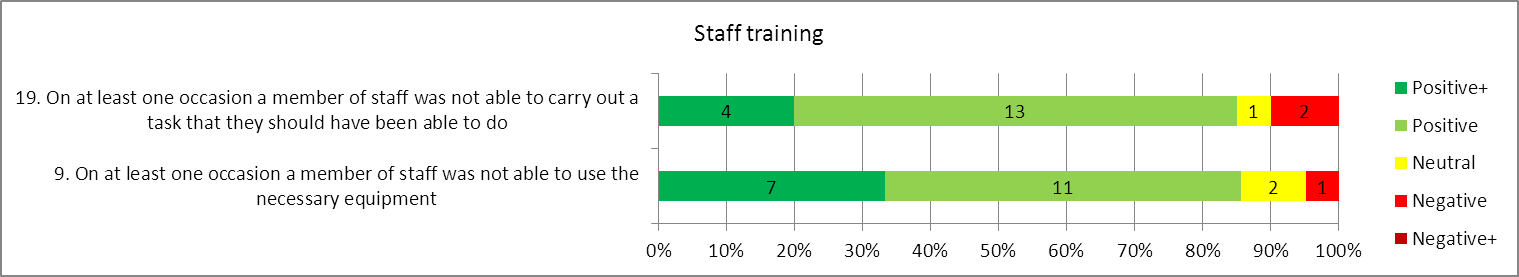


No patients reported a safety concern or experience related to any of the above questions

No specific general comments were received that related to the above questions

No specific positive comments were received that related to the above questions

**8. Equipment (design and functioning)**

**
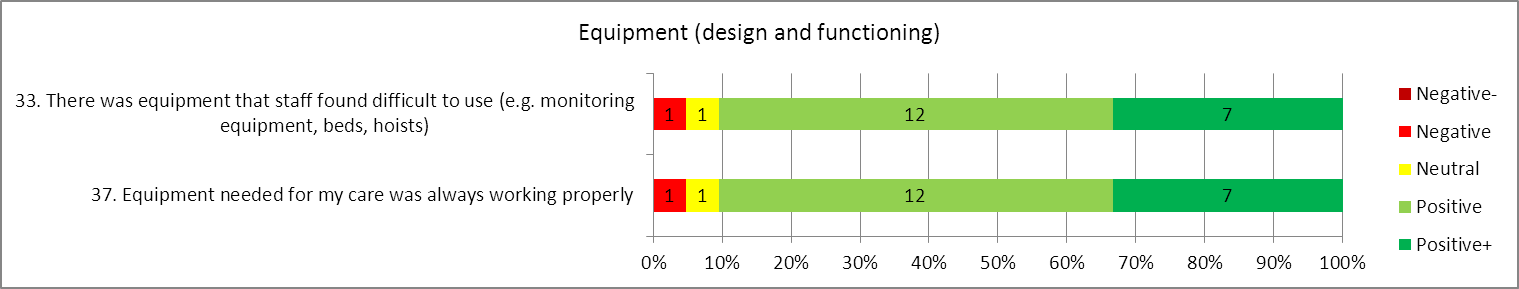
**

No patients reported a safety concern or experience related to any of the above questions

**What other general comments did patients make in relation to the questions about equipment?**

| **Question No.** | **Question Text** | **Patient Comment** |
| --- | --- | --- |
| 37 | Equipment needed for my care was always working properly | *Just a few niggles with beds and chairs.* |
| 33 | There was equipment that staff found difficult to use (e.g. monitoring equipment, beds, hoists) | *A chair was rickety and she had a job to sort it.* |

9. **Delays**

No specific positive comments were received that related to the above questions

**
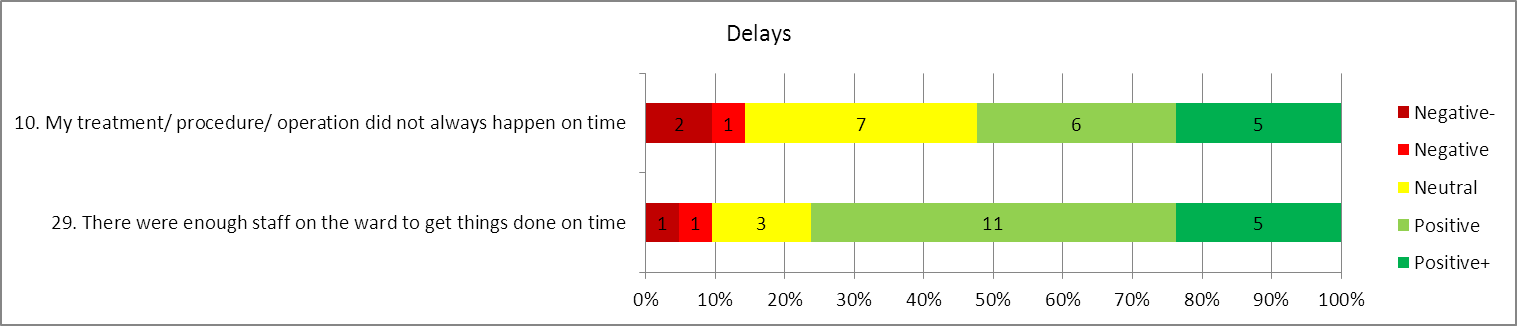
**

**What patient reported safety concerns were linked to questions about delays?**

| **Question No.** | **Question Text** | **What happened / why did it happen?** | **What can be done to prevent it happening again?** | **Patient-rated preventability** | **Patient-rated severity** |
| --- | --- | --- | --- | --- | --- |
| 10 | My treatment/procedure/operation did not always happen on time | *The team required to anaesthetise me for a shoulder procedure was not available immediately (they had attempted it with gas and air) so I had to wait another day. Caused more pain for longer.* | *I don't know* | Don’t know | 10 |
|  |  | *They rang me up at home on Tuesday so I had to be in by 7.30 Wednesday morning, and then didn't go down until 3.30 - a long wait but they were all very kind. It's a system they have to work on.* |  |  |  |

**What other general comments did patients make in relation to the questions about delays?**

| **Question No.** | **Question Text** | **Patient Comment** |
| --- | --- | --- |
| 29 | There were enough staff on the ward to get things done on time | *Enough staff but they have so much paperwork to deal with, that sometimes they can't get round to patient care.* |
| 10 | My treatment/procedure/operation did not always happen on time | *Non-urgent case so repeatedly cancelled (3 times).* |

No specific positive comments were received that related to the above questions

**10. Any other general comments, positive experiences of care, or patient reported safety concerns that were not related to any specific questions**

**What safety concerns did patients report not related to a specific question?**

| **What happened / why did it happen?** | **What can be done to prevent it happening again?** | **Patient-rated preventability** | **Patient-rated severity** |
| --- | --- | --- | --- |
| *No washing facilities were offered in the morning, had to ask for them. Also no hand washing facilities were offered after patient had used the bedpan. Whilst making this report, the patient overheard the patient in the next bed also asking for help with washing. Do not feel clean, and want to wash and brush teeth. It is un-hygienic not to be able to wash hands after using bedpan.* | *Offer wash bowls and help to all patients who need it. Do not wait for them to ask. Offer hand washing facilities after bedpan use.* | Definitely yes | 7 |
| *Worried mainly about after-care. I will have to go to bed when the home care team come, not when convenient to me. I understand why this is the case but it's not great.* | *I have been an engineer all my life and I am sure there must be a way of adapting a piece of equipment to help me pull up covers so I can do it myself.* | Probably yes | 4 |

No patients reported any general additional comments

**What positive experiences of care did patients report that were not related to a specific question?**

- Everything that has happened to me since I got here has been brilliant.
- Generally friendly attitude, especially the domestic staff.
- Generally the staff are so conscientious and always willing to help.
- Baby type mugs to hold hot tea - they cool them down

Appendix 5. Staff safety culture questionnaire

**PRASE (Patient Reporting and Action for a Safe Environment) STAFF QUESTIONNAIRE**

As part of our PRASE research project we are hoping to compare the views of staff and patients in the same ward. It is really important that we get as many staff as possible on each participating ward to fill in this brief questionnaire.

The surveys are anonymous, but we do ask for some background general information on the last page. This will help us in the analysis of the questionnaire.

**Section 1: General attitudes**

Please complete the survey for your current job, or the job you do most of the time. If you work across multiple wards in the hospital please answer in relation to the ward where you were given this questionnaire.

Please read each question carefully, but give your immediate response by ticking the box which best matches your personal view.

| 1. To what extent do you agree or disagree with each of the following statements about working in your ward | Strongly disagree | Disagree | Neither agree nor disagree | Agree | Strongly agree |
| --- | --- | --- | --- | --- | --- |
| 1. I would recommend my ward as a place to work. | _1_ | _2_ | _3_ | _4_ | _5_ |
| 1. If a friend or relative needed treatment, I would be happy with the standard of care provided by this ward. | _1_ | _2_ | _3_ | _4_ | _5_ |

| 2. To what extent do you agree or disagree with each of the following statements about your contribution | Strongly disagree | Disagree | Neither agree nor disagree | Agree | Strongly agree |
| --- | --- | --- | --- | --- | --- |
| 1. I am satisfied with the quality of care I give to patients / service users. | _1_ | _2_ | _3_ | _4_ | _5_ |
| 1. I feel that my role makes a difference to patients / service users. | _1_ | _2_ | _3_ | _4_ | _5_ |
| 1. I am able to deliver the patient care I aspire to. | _1_ | _2_ | _3_ | _4_ | _5_ |

| 3. To what extent do you agree or disagree with each of the following statements about improving work practices | Strongly disagree | Disagree | Neither agree nor disagree | Agree | Strongly agree |
| --- | --- | --- | --- | --- | --- |
| 1. I am able to make suggestions to improve the work of my team / department. | _1_ | _2_ | _3_ | _4_ | _5_ |
| 1. There are frequent opportunities for me to show initiative in my role. | _1_ | _2_ | _3_ | _4_ | _5_ |
| 1. I am able to make improvements happen in my area of work. | _1_ | _2_ | _3_ | _4_ | _5_ |
| 1. Health care professionals and managers in non-clinical roles work well together in my area of work. | _1_ | _2_ | _3_ | _4_ | _5_ |
| 1. Senior managers act on staff feedback | _1_ | _2_ | _3_ | _4_ | _5_ |

**Section 2: Your work area / unit**

**Please indicate your agreement or disagreement with the following statements about your work area/unit.**

|  | **Strongly disagree** | **Disagree** | **Neither** | **Agree** | **Strongly agree** |
| --- | --- | --- | --- | --- | --- |
| 4. It is just by chance that more serious mistakes don’t happen around here | 🞎1 | 🞎2 | 🞏3 | 🞎4 | 🞏5 |
| 5. Patient safety is never sacrificed to get more work done | 🞎1 | 🞎2 | 🞏3 | 🞎4 | 🞏5 |
| 6. We have patient safety problems in this unit | 🞎1 | 🞎2 | 🞏3 | 🞎4 | 🞏5 |
| 7. Our procedures and systems are good at preventing errors from happening | 🞎1 | 🞎2 | 🞏3 | 🞎4 | 🞏5 |

**Section 3: Frequency of events reported**

|  | **Never** ⯆ | **Rarely** ⯆ | **Some-times** ⯆ | **Most of the time** ⯆ | **Always** ⯆ |
| --- | --- | --- | --- | --- | --- |
| 8. When a mistake is made, but is *caught and corrected before affecting the patient*, how often is this reported? | 🞎1 | 🞎2 | 🞏3 | 🞎4 | 🞏5 |
| 9. When a mistake is made, but has *no potential to harm the patient*, how often is this reported? | 🞎1 | 🞎2 | 🞏3 | 🞎4 | 🞏5 |
| 10.When a mistake is made that *could harm the patient*, but does not,  how often is this reported? | 🞎1 | 🞎2 | 🞏3 | 🞎4 | 🞏5 |

**In your hospital work area/unit, when the following mistakes happen, *how often are they reported?***

**Section 4: Patient Safety Grade**

**11. Please give your work area/unit in this hospital an overall grade on patient safety.**

| 🞎 | 🞎 | 🞎 | 🞎 | 🞎 |
| --- | --- | --- | --- | --- |
| **A**  Excellent | **B**  Very Good | **C**  Acceptable | **D**  Poor | **E**  Failing |

**Section 5: Number of events reported**

**12. In the past 12 months, how many event reports have you filled out and submitted?**

| 🞎 a. No event reports | 🞎 d. 6 to 10 event reports |
| --- | --- |
| 🞎 b. 1 to 2 event reports | 🞎 e. 11 to 20 event reports |
| 🞎 c. 3 to 5 event reports | 🞎 f. 21 event reports or more |

**SECTION 6: Background Information**

**This information will help in the analysis of the survey results.**

**13a. What is your job title?.............................................................................................................**

**13b. What is your primary work area or unit?..............................................................................**

**14. How long have you worked in your current hospital work area/unit?**

| 🞎 a. Less than 1 year | 🞎 d. 11 to 15 years |
| --- | --- |
| 🞎b. 1 to 5 years | 🞎 e. 16 to 20 years |
| 🞎 c. 6 to 10 years | 🞎 f. 21 years or more |

**15. PLEASE ENTER TODAY’S DATE: …………………………………………………………………**

Appendix 6. PRASE INTERVENTION & PROCESS EVALUATION: TRIAL SUMMARY DIAGRAM

Patient experience of safety measured, Phase 1

(25 patients per ward)

‘Start-up session’ with APTs (1 per Trust)

Patient experience of safety measured, Phase 2

Patient experience of safety measured, Phase 3

Final follow-up meeting held within each trust

Wards randomised to intervention or control group

INTERVENTION GROUPS

CONTROLS

Wards identify Action Planning Teams (APTs)

Wards receive first feedback report

APTs action plan, implement and monitor changes

APTs action plan, implement and monitor changes

Wards receive second feedback report

Control wards receive feedback report with information from all three measurement periods

Wards receive final feedback report

No further action planning required

6-month ‘update’ session with APTs

1. **Reflective diaries** **1**

**B) Ethnographic**

**observation of APMs 1**

**C) Interviews with AP leads (1)** (telephone) before receipt of 2^nd^ report

1. **Reflective diaries** **2**

**B) Ethnographic**

**observation of APMs 2**

**C) Interviews with AP leads (2)** (telephone) before receipt of final report

1. **Reflective diaries** **3**

**D) Questionnaire to(all staff**

Appendix 7. Intervention fidelity assessment table

| **Intervention component** | **Component to be assessed?** | **Adherence components**  Comprising (where appropriate¹:  i) Content  ii) Frequency/ duration  iii) Reach | **Adherence assessment** | **Potential moderating factors²** |
| --- | --- | --- | --- | --- |
| Orientation meeting and brief training about organisational models of safety and format of the intervention for the lead in each unit | YES | Content  Reach (Target group: PRASE Ward leads and ward staff) | No adherence 0  Some adherence - 1  Mostly adhering - 2  Full adherence - 3 | Context (including management support)  Responsiveness  Quality of delivery |
| Collection of feedback from patients about the safety of their care environment | NO - RESEARCHER CONTROLLED |  |  |  |
| Collection of feedback from patients about safety incidents and positive experiences of care | NO - RESEARCHER CONTROLLED |  |  |  |
| Reporting collated feedback to the lead of the unit | NO - RESEARCHER CONTROLLED |  |  |  |
| Holding a multi-disciplinary action planning meeting to consider feedback | YES | Content  Reach (Target group: multi-professional / multi-level representation from ward) | No adherence - 0  Some adherence - 1  Mostly adhering - 2  Full adherence - 3 | Context (including management support)  Responsiveness  Quality of delivery |
| Facilitation of the action planning meeting. | NO - RESEARCHER CONTROLLED |  |  |  |
| Creation of action plans in response to feedback | YES | Content | No adherence - 0  Some adherence - 1  Mostly adhering - 2  Full adherence - 3 | Context (including management support)  Responsiveness  Quality of delivery |
| Implementation of the action plans | YES | Content | No adherence - 0  Some adherence - 1  Mostly adhering - 2  Full adherence - 3 | Context (including management support)  Responsiveness  Quality of delivery |
| ‘Mid-point’ meeting to share ideas and to consider progress | YES | Content  Reach (Target group: PRASE Ward leads and ward staff) | No adherence - 0  Some adherence - 1  Mostly adhering - 2  Full adherence - 3 | Context (including management support)  Responsiveness  Quality of delivery |
| ‘Closing’ session to summarise progress and lessons learned | YES | Content  Reach (Target group: PRASE Ward leads and ward staff) | No adherence - 0  Some adherence - 1  Mostly adhering - 2  Full adherence - 3 | Context (including management support)  Responsiveness  Quality of delivery |

^1^Following Hasson *et al.* (2010), adherence assessed using:

i) Content - was the intervention component implemented as planned?

ii) Reach - what proportion of the target group participated in this intervention component?

Hasson *et al.* (2010) third component ‘Frequency/ duration’ dropped for this study as each of the components represents a one off event so frequency/duration would not be an appropriate adherence criterion to assess (Frequency/ duration - was the intervention component implemented as often and for as long as planned?)

^2^Following Hasson *et al.* (2010), only three potential moderating factors were selected from their list, as the others were deemed to be under the control of the researchers within the study (for example, recruitment of participants, comprehensiveness of policy description). These moderators will be assessed through qualitative methods.

i) Context (including management support) - What factors at political, economic, organizational, and work group levels affected the implementation?

ii) Responsiveness - How were the participants engaged with the intervention component? How satisfied were the participants with the intervention component? How did the participants perceive the outcomes and relevance of the intervention component?

iii) Quality of delivery - How was the quality of delivering the intervention components?
